# Perioperative Hyperhidrosis: Case Reports of Two Patients and Literature Review

**DOI:** 10.7759/cureus.79370

**Published:** 2025-02-20

**Authors:** Yassine Ben Saad, Muhammad Jaffar Khan, Arunabha Karmakar, Muhammad Firas Alhammad, Muhammad Yousaf, Wajeeha Arshad, Moncef Ben Ghoulem Ben Saad

**Affiliations:** 1 Internal Medicine, UCLan School of Medicine &amp; Dentistry, University of Central Lancashire, Preston, GBR; 2 Anesthesiology and Critical Care, Hamad Medical Corporation, Doha, QAT

**Keywords:** case report, general anesthesia, hyperhidrosis, perioperative care, sweating

## Abstract

Perioperative hyperhidrosis (POH) can present as excessive sweating within hours after anesthetic induction and may persist up to 24 hours postoperatively following general anesthesia. While commonly a benign finding, excessive or persistent POH can be disconcerting for the patient and can occasionally signify an underlying infection, electrolyte disturbances, or autonomic dysregulation.

A systematic approach to managing POH has not been published. We report the cases of two patients with excessive sweating under general anesthesia, highlighting their clinical presentation and management. We also propose a clinical algorithm to standardize the management of POH.

## Introduction

Hyperhidrosis is characterized by excessive sweating, with a global incidence ranging from 2.5% to 18% [1]. However, perioperative sweating remains poorly studied, and its exact incidence is yet to be determined. Perioperative hyperhidrosis (POH), though not extensively documented, has been observed in various surgical contexts. A study focusing on patients undergoing elective cardiac surgery reported that 46% of participants experienced postoperative sweating, with 22% experiencing mild sweating and 24% experiencing moderate and profuse sweating [2]. POH can be mild or excessive, localized or generalized, self-limited or persistent, benign or concerning, or physiological or pathological/iatrogenic. However, the exact occurrence of POH depends on the patient population, type of surgery, and anesthetic technique. Notably, the World Health Organization - International Classification of Disease registry does not include POH as a distinct clinical entity, which may limit its reporting [3]. Perioperative sweating has been reported in multiple case studies, occurring both intraoperatively and postoperatively, with potential contributing factors such as physiological stress, metabolic imbalances, autonomic dysfunction, and the effects of anesthetic drugs, with symptoms persisting for up to 24 hours postoperatively [3-6]. Intraoperative sweating has been reported in patients who experience significant temperature dysregulation during surgery or a high-stress response during anesthesia. While most cases of POH are benign and self-limiting, they are sufficiently prevalent and alarming to warrant patient assessment before clearance for or discharge from surgery.

We report the cases of two patients with POH that developed during emergence from general anesthesia and tracheal extubation, persisted in the postanesthesia care unit (PACU), and was associated with hypothermia. We describe our clinical observations, correlate the findings with the literature, and provide insights into the mechanisms and potential causes of sweating in the perioperative period.

## Case presentation

Patient 1

A 33-year-old male with ASA physical status 2, obesity (weight 105 kg, BMI 31.18 kg/m^2^), and a history of type 2 diabetes mellitus was scheduled for elective incisional hernia repair. He had a previous history of sleeve gastrectomy, which was complicated by thrombosis of the superior mesenteric vein and portal vein, leading to bowel ischemia and necessitating laparotomy. He was receiving 20 mg of rivaroxaban daily for anticoagulation, which was held 48 hours before his scheduled surgery, and metformin for blood glucose control.

**Figure 1:**
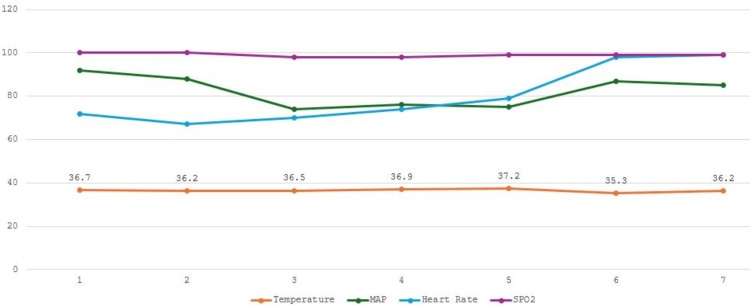
Perioperative vitals of the first patient. The x-axis is marked with seven points representing various time periods during the surgery when the patient's vitals were recorded: 1, preoperative (baseline line) vitals; 2, 5 minutes post-induction of general anesthesia; 3, first hour post-induction; 4, second hour post-induction; 5, vitals at emergence; 6, vitals at PACU admission; and 7, one hour after PACU admission. MAP, mean arterial pressure; PACU, postanesthesia care unit; SPO_2_, oxygen saturation

On the day of surgery, the patient was stable preoperatively. Anesthesia was induced intravenously with 200 mg (2 mg/kg) propofol, 200 mcg (2 mcg/kg) fentanyl, and 50 mg (0.5 mg/kg) rocuronium. Anesthesia was maintained with inhaled sevoflurane and intravenous (IV) remifentanil infusion. Active warming device was applied to achieve normothermia. Other intraoperative medications included IV ketorolac (30 mg), dexamethasone (8 mg), acetaminophen (1 gm), and morphine (10 mg). He was infused with 2000 mL of crystalloids (Ringer’s lactate) and 250 mL of colloids (purified protein fraction 5%). The patient was vitally stable intraoperatively and normothermic (Figure 1).

Emergence from anesthesia and tracheal extubation were uneventful but were associated with profuse sweating. Post-emergence, the patient was alert, hemodynamically stable, and reported no pain but continued to sweat profusely and became hypothermic without shivering. In the PACU, his body temperature was recorded as 35.3°C. The serum glucose level was normal.

He was treated with active air warming and draped with a sheet. Sweating continued for one and a half hours and then subsided gradually. As the patient was vitally stable and reported no pain, he was transferred to the ward for continuing care.

Patient 2

A 55-year-old male with an ASA physical status of 2 was scheduled for laparoscopic cholecystectomy. He had hypertension, which was well controlled with amlodipine, and type 2 diabetes mellitus, which was well controlled with metformin.

**Figure 2:**
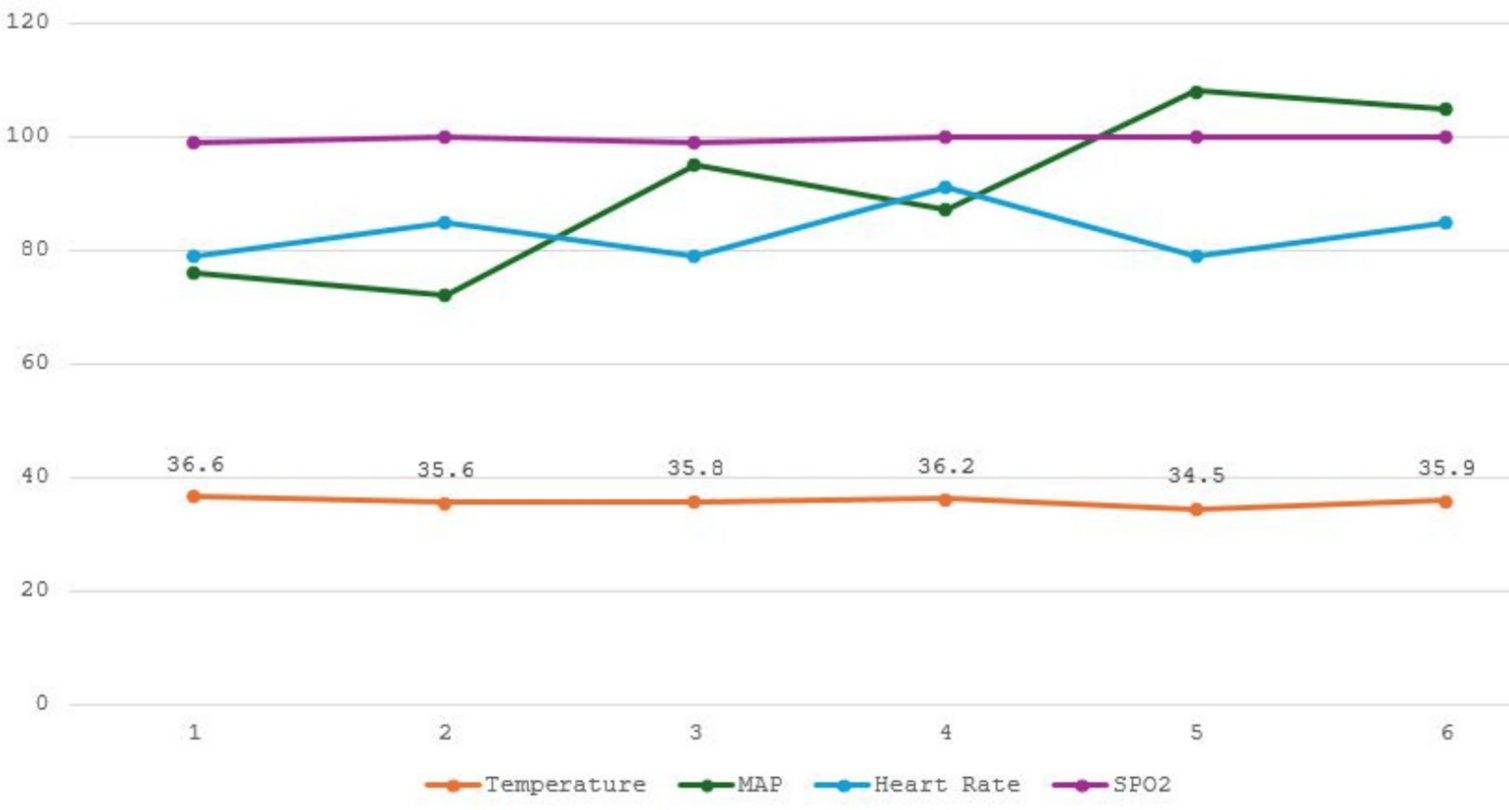
Perioperative vitals for the second case. The x-axis is marked with six points representing various time periods during the surgery when the patient’s vitals were recorded: 1, preoperative (baseline line) vitals; 2, 5 minutes post-induction of general anesthesia; 3, first hour post-induction; 4, vitals at emergence; 5, vitals at the admission to PACU; and 6, one hour after admission to the PACU. MAP, mean arterial pressure; PACU, postanesthesia care unit; SPO_2_, oxygen saturation

On the day of surgery, anesthesia was induced intravenously with propofol 150 mg (2 mg/kg), fentanyl (2 mcg/kg), and rocuronium (0.6 mg/kg) and maintained with sevoflurane inhalation. Additionally, the patient received IV dexamethasone 8 mg, ketorolac 30 mg, acetaminophen 1 gm, and morphine 10 mg for analgesia. He received one liter of Ringer’s lactate. His body temperature was maintained with active warming. Toward the end of the procedure, sweating was observed on his face and arms, despite a normal body temperature of 36.2°C. Active warming was discontinued, and the patient was transferred to the PACU after emergence and extubation. The perioperative vital signs are shown in Figure 2.

The patient’s body temperature in the PACU was 34.5°C, with continued sweating. The random blood sugar concentration was 10.4 mmol/L. Active air warming was utilized to normalize his temperature. The sweating continued for one hour and then subsided.

## Discussion

Our patients had persistent POH with postoperative hypothermia after otherwise uneventful general anesthesia. Both had recovered with expectant management. When excessive sweating occurs during emergence, it is essential to assess the patient's overall clinical status, hemodynamics, pain levels, medication effects, and body temperature. Potential interventions include pharmacological measures such as anticholinergic agents to reduce sweat gland activity or active warming techniques to counteract hypothermia [6]. However, in both our cases, they were managed conservatively, and the patients’ condition improved spontaneously.

Common causes of POH include covering patients with surgical drapes, surgical stimulation, autonomic dysregulation, medication-related effects, hypoglycemic episodes, or other underlying causes [7,8].

Human eccrine and apocrine sweat glands are involved in sweating. Eccrine glands are present throughout the body's skin surface and are the predominantly active sweat glands. In contrast, apocrine glands become active after puberty when stimulated by hormonal changes. Both glands have sympathetic innervation mediated adrenergically and are involved primarily in emotional sweating triggered by stress, fear, and pain [9,10].

The hypothalamic thermal center is responsible for regulating body temperature and receives stimuli from temperature-sensing nerves or directly senses the temperature of the blood. Additionally, localized sweating centers have been identified in the thoracic and lumbar regions [9]. Sweating due to increased body temperature is known as thermal sweating, which serves the primary purpose of heat dissipation through evaporation. It is reflexive and can precede a rise in body temperature when exposed to external heat. Thermal sweating tends to be generalized throughout the body. Physiologically, if one area of the body is warm, sweating can occur across the entire body.

Sweating can also be triggered by autonomic activity through sympathetic (mediated by adrenaline and noradrenaline) or parasympathetic activation (mediated by acetylcholine). While the primary innervation of sweat glands is cholinergic, adrenaline and noradrenaline also play a role [9,11]. An individual's specific autonomic response, whether sympathetic or parasympathetic, cannot be accurately predicted in advance. Typically, an increase in heart rate and blood pressure signifies a sympathetic response, whereas a decrease in blood pressure and pulse rate, accompanied by cold sweat, indicates a parasympathetic response. Under general anesthesia, both sympathetic and parasympathetic-mediated sweating can occur [11,12]. Sympathetic discharge can result from anxiety, pain, or hypercarbia in the presence of inadequate anesthesia. Sweating may also be accompanied by bradycardia, nausea, and hypotension as part of a generalized vagal reaction or as a thermoregulatory response to hyperthermia. During surgical stimulation, in the lighter plane of anesthesia, an enhanced sympathetic response can cause an increase in blood pressure and pulse rate, with or without sweating, but sweating is mostly induced by sympathetic system stimulation. Both hypoxia and hypercarbia can lead to increased blood pressure and sweating [13]. Hemorrhage and pain may elicit a parasympathetic response, resulting in cold sweat [11,13].

Sweating can also be caused by multiple drugs that the patient may be taking or has received intraoperatively. Special attention is warranted for patients taking antidepressants, as sweating can be indicative of serotonin syndrome, which is typically accompanied by high blood pressure and tachycardia. Patients who are taking selective serotonin reuptake inhibitors (SSRIs) along with pain medications such as tramadol, meperidine, and fentanyl, and anti-nausea medications such as ondansetron or migraine medications may be at risk of developing serotonin syndrome. Other drugs that contribute to perioperative sweating are nonsteroidal anti-inflammatory drugs (NSAIDs), beta-agonists, proton pump inhibitors, steroids, oral hypoglycemic drugs, and triptans [8,13,14].

Opioid-induced sweating has been well documented [4,15]. Specifically, two cases have been reported in which patients who underwent spinal anesthesia with intrathecal morphine injections experienced excessive sweating and hypothermia [5,16]. The regulation of body temperature involves multiple opioid receptors in the hypothalamus, and the distribution of spinal opioids may interfere with the temperature set point [4]. Furthermore, opioids such as morphine can stimulate mast cells, a type of immune cell, leading to the release of histamine, which can cause skin warmth and sweating [15]. Excessive sweating has also been associated with the use of oral methadone and transdermal fentanyl, possibly again attributed to opioid-induced mast cell release of histamine [8,17]. Similarly, patients may experience sweating after receiving remifentanil, a potent opioid analgesic [6].

Hypoglycemia can also cause sweating, and blood sugar must be monitored in diabetic patients who are under anesthesia. Other potential causes of sweating include vitamin D deficiency or hyperthyroidism. In addition to medication-related and preexisting disease causes, excessive warming and draping during procedures can result in sweating [18].

Cold-induced sweating syndrome is a genetic disorder inherited in an autosomal recessive manner. It is characterized by dysmorphic features and sweats on the face, arms, and posterior chest when exposed to temperatures below 18-22°C [19]. Nervousness can also trigger sweating in individuals with this condition.

Notable rare or serious clinical pathologies causing POH have also been described. Shapiro syndrome is a rare condition characterized by recurrent hypothermia, sweating, and chills due to agenesis of the corpus callosum [20]. Pheochromocytoma is a tumor of the adrenal glands characterized by excessive cortisol release that presents with hypertension and a triad of diaphoresis, headaches, and palpitations [21]. In patients with pheochromocytoma, the release of adrenaline can lead to significant sweating. Similarly, familial dysautonomia (FD) is a rare genetic disorder marked by autonomic dysfunction, recurrent pulmonary infections, esophageal dysmotility, spinal abnormalities, and episodic dysautonomic crises. These crises present with symptoms such as rash, vomiting, sweating, and hypertension. Patients with FD are prone to frequent anesthetic complications [22].

**Figure 3:**
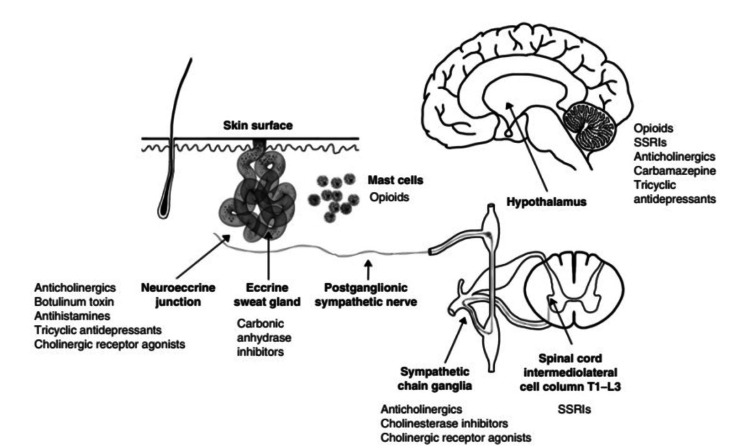
Schematic representation showing the major sites of action of classes of drugs that commonly influence sweating. The sweating pathway begins in the hypothalamus and extends to the intermediolateral column, from which arise preganglionic sympathetic fibers that leave the spinal cord and synapse within the sympathetic chain ganglia. Postganglionic sympathetic nerves emerge from these ganglia and travel alongside arteries to reach their subdermal targets, the eccrine sweat glands. Drugs can interact with each level of this pathway. Reproduced with permission from [4]. SSRI, selective serotonin reuptake inhibitor

Various strategies can be employed to mitigate sweating based on the cause. These include measures to prevent hyperthermia, maintain the depth of anesthesia, regulate blood sugar, ensure oxygenation and ventilation, address any underlying metabolic conditions, and mitigate sympathetic stimuli. Vasopressors may be administered to maintain blood pressure, and supplemental fluid may be required for normovolemia and to maintain adequate cardiac output (Figure 3).

**Figure 4:**
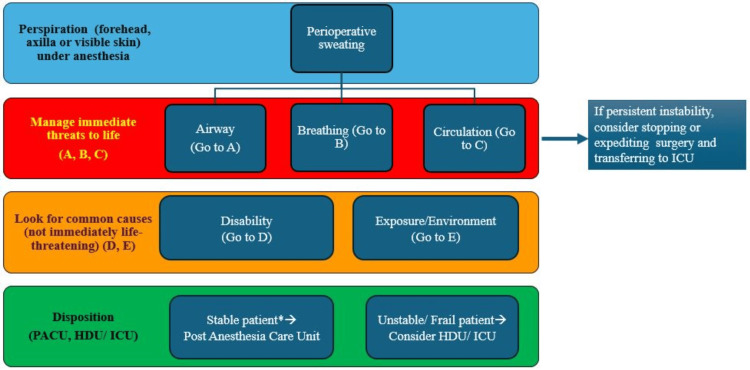
Suggested algorithm to approach perioperative sweating (continued). *Stability refers to cardiopulmonary stability. Continued sweating in an otherwise stable patient can still be observed in the PACU. HDU, high dependency unit; ICU, intensive care unit; PACU, postanesthesia care unit

**Figure 5:**
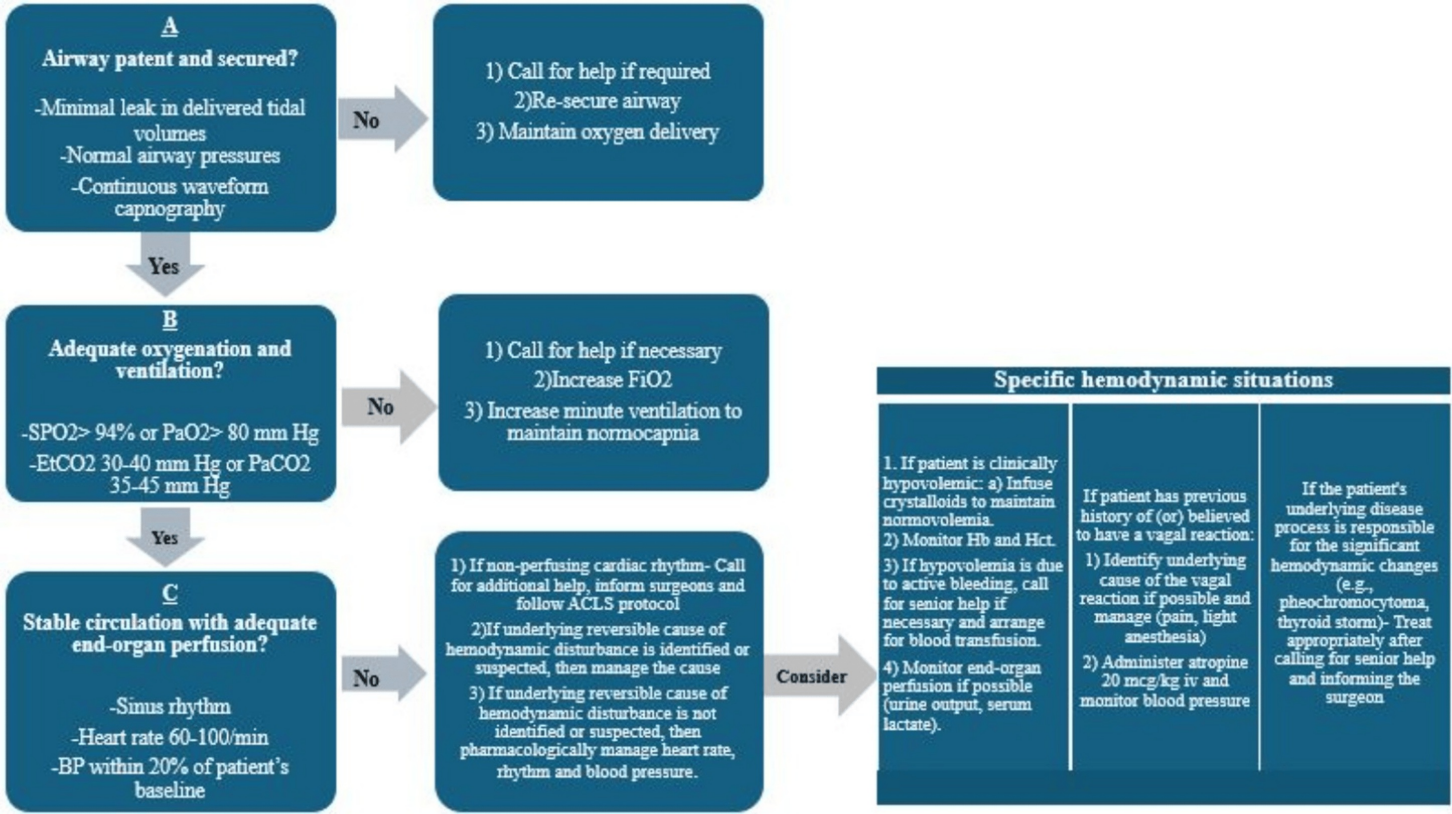
Suggested algorithm to approach perioperative sweating (continued). ACLS, advanced cardiac life support; EtCO_2_, end tidal carbon-dioxide; FiO_2_, fraction of inspired oxygen concentration; Hb, hemoglobin; Hct, hematocrit; iv, intravenous; kg, kilogram; mcg, microgram; PaCO_2_, arterial partial pressure of carbon-dioxide; PaO_2_, arterial partial pressure of oxygen; SPO_2_, oxygen saturation

**Figure 6:**
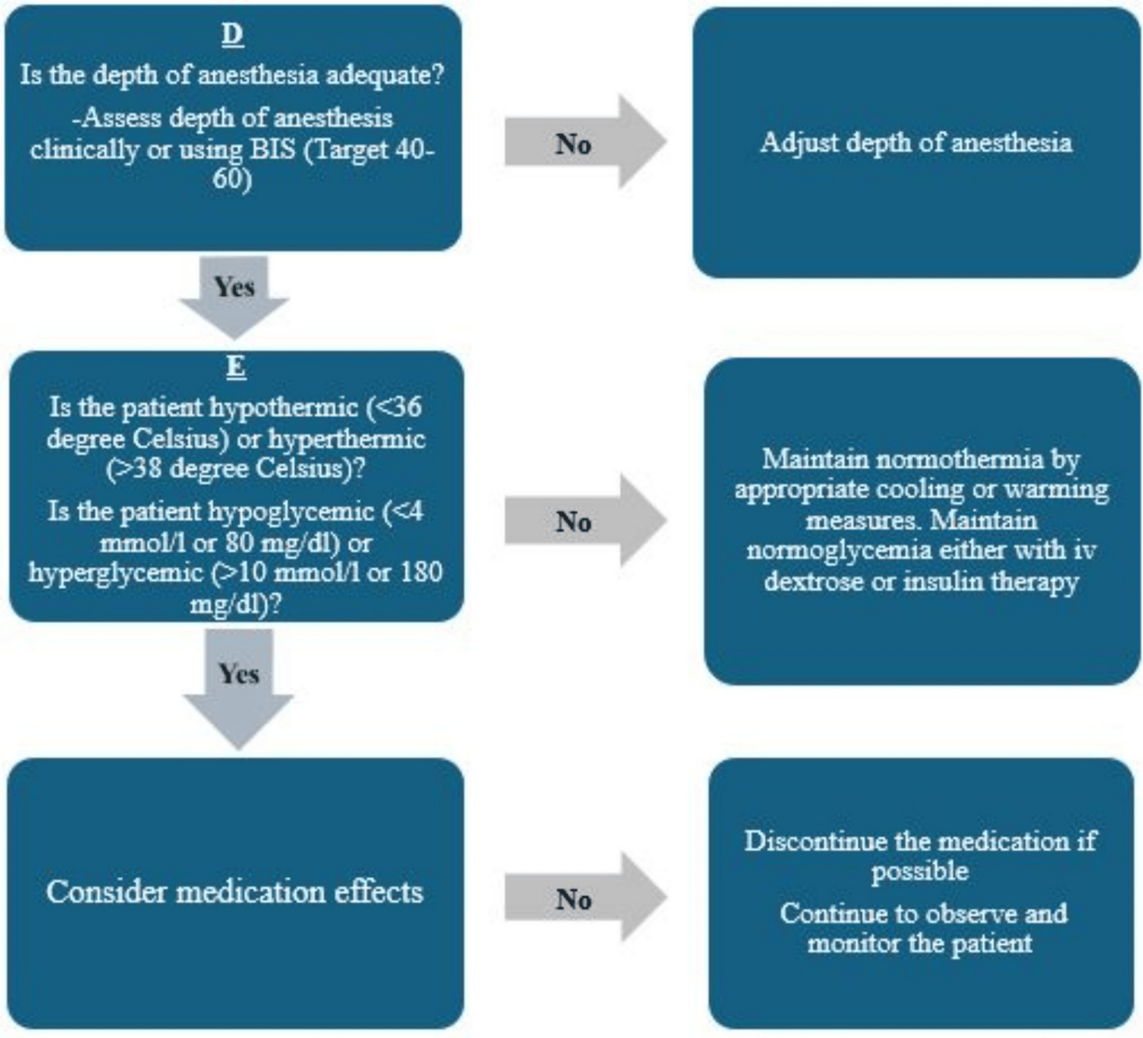
Suggested algorithm to approach perioperative sweating. BIS, bispectral index; iv, intravenous

As POH may be a red flag for an underlying physio-pathological condition, a stepwise approach helps rule out significant pathologies, deliver appropriate care, and mitigate intraoperative mortality. The algorithm in Figures 4-6 is a suggested approach proposed by the authors on the basis of their experience.

Both patients exhibited stable hemodynamics, maintained adequate oxygen saturation, and had normal end-tidal carbon dioxide levels intraoperatively. During a follow-up assessment three months later, both patients were reported to be in good health, with no complications or concerns related to their perioperative management.

## Conclusions

The exact etiology of POH in our patients was not identified. It is plausible that a combination of active warming and medications such as steroids, morphine, and NSAIDs may have contributed. Remarkable features of POH in our patients included its persistence for 60-90 minutes postoperatively, associated hypothermia, and self-resolution.

POH is caused by various factors, such as surgical stimulation, opioid administration, or alterations in the autonomic nervous system associated with aging. It is crucial to promptly evaluate the underlying causes of excessive sweating during anesthesia, as it could indicate a serious underlying medical issue or potentially lead to complications on its own. Timely assessment and identification of the root cause are essential for ensuring appropriate management and intervention to address sweating and mitigate any associated risks or complications. If the patient is otherwise fit, they may recover with expectant management.

## References

[1] Oshima Y, Fujimoto T, Nomoto M, Fukui J, Ikoma A (2023). Hyperhidrosis: a targeted literature review of the disease burden. J Dermatol.

[2] Mathew A, Mathew M, Balamurugan B (2022). Awareness and sweating among patients undergoing elective cardiac surgery under hypothermic cardiopulmonary bypass. MedPulse Int J Anesthesiol.

[3] (2025). International Statistical Classification of Diseases and Related Health Problems 10th Revision. https://icd.who.int/browse10/2019/en.

[4] Ferraz S, Caria T, Da Silva AV, Candeias MJ, Cenicante T (2018). Persistent hypothermia and excessive sweating following intrathecal morphine administration in a teenage boy: a case report. Anesth Pain Med.

[5] Sayyid SS, Jabbour DG, Baraka AS (2003). Hypothermia and excessive sweating following intrathecal morphine in a parturient undergoing cesarean delivery. Reg Anesth Pain Med.

[6] Kim H, Jee D, Lee H (2015). Unusual excessive sweating and hypothermia during hysterectomy under general anesthesia: a case report. Anesth Pain Med.

[7] Wörle B, Rapprich S, Heckmann M (2007). Definition and treatment of primary hyperhidrosis. J Dtsch Dermatol Ges.

[8] Cheshire WP, Fealey RD (2008). Drug-induced hyperhidrosis and hypohidrosis: incidence, prevention and management. Drug Saf.

[9] Baker LB (2019). Physiology of sweat gland function: the roles of sweating and sweat composition in human health. Temperature (Austin).

[10] Cramer MN, Gagnon D, Laitano O, Crandall CG (2022). Human temperature regulation under heat stress in health, disease, and injury. Physiol Rev.

[11] Sessler DI (2016). Perioperative thermoregulation and heat balance. Lancet.

[12] Schick CH (2016). Pathophysiology of hyperhidrosis. Thorac Surg Clin.

[13] Linton CD (1961). Sweating and anesthesia. A consideration of causes and effects. Anesthesiology.

[14] De Witte J, Sessler DI (2002). Perioperative shivering: physiology and pharmacology. Anesthesiology.

[15] Mazy A (2016). Excessive sweating following intrathecal μ agonists: effective atropine management. Egypt J Anaesth.

[16] Serhat K, Eraydin G (2023). #36365 Miraculous treatment of excessive sweating associated with intrathecal morphine: case report. BMJ.

[17] Nelson L, Schwaner R (2009). Transdermal fentanyl: pharmacology and toxicology. J Med Toxicol.

[18] John M, Ford J, Harper M (2014). Peri-operative warming devices: performance and clinical application. Anaesthesia.

[19] Hahn AF, Knappskog PM (2024). Cold-induced sweating syndrome including Crisponi syndrome. GeneReviews® [Internet].

[20] Boskovic S, Ciobanu V, Theuerkauf N, Bakhtiary F, Velten M (2023). Case report: Perioperative management of a patient with shapiro syndrome during on-pump cardiac surgery. Front Cardiovasc Med.

[21] Harrison A, Tashdjian C, Rampal M (2023). A case of generalized, unrelenting sweating resulting in social isolation for over two decades. Cureus.

[22] Weingarten TN, Sprung J, Burgher AH (2007). Perioperative management of familial dysautonomia: a systematic review. Eur J Anaesthesiol.

